# Effect of Selected Plant Phenolics on Fe^2+^-EDTA-H_2_O_2_ System Mediated Deoxyribose Oxidation: Molecular Structure-Derived Relationships of Anti- and Pro-Oxidant Actions

**DOI:** 10.3390/molecules22010059

**Published:** 2016-12-31

**Authors:** Jeffrey de Graft-Johnson, Dariusz Nowak

**Affiliations:** 1 Heart and Vascular Institute of North Florida, 2623 Centennial Blvd., Suite 102, Tallahassee, FL 32308, USA; 2 Department of Clinical Physiology, Medical University of Lodz, Mazowiecka 6/8, 92-215 Lodz, Poland; dariusz.nowak@umed.lodz.pl

**Keywords:** hydroxyl radicals, polyphenols, plant phenolic acids, Fenton system

## Abstract

In the presence of transition metal ions and peroxides, polyphenols, well-known dietary antioxidants, can act as pro-oxidants. We investigated the effect of 13 polyphenols and their metabolites on oxidative degradation of deoxyribose by an ^•^OH generating Fenton system (Fe^2+^-ethylenediaminetetraacetic acid (EDTA)-H_2_O_2_). The relationship between phenolics pro-oxidant/anti-oxidant effects and their molecular structure was analyzed using multivariate analysis with multiple linear regression and a backward stepwise technique. Four phenolics revealed a significant inhibitory effect on OH-induced deoxyribose degradation, ranging from 54.4% ± 28.6% (3,4-dihydroxycinnamic acid) to 38.5% ± 10.4% (catechin) (*n* = 6), correlating with the number of –OH substitutions (*r* = 0.58). Seven phenolics augmented the oxidative degradation of deoxyribose with the highest enhancement at 95.0% ± 21.3% (quercetin) and 60.6% ± 12.2% (phloridzin). The pro-oxidant effect correlated (*p* < 0.05) with the number of –OH groups (*r* = 0.59), and aliphatic substitutes (*r* = −0.22) and weakly correlated with the occurrence of a catechol structure within the compound molecule (*r* = 0.17). Selective dietary supplementation with phenolics exhibiting pro-oxidant activity may increase the possibility of systemic oxidative stress in patients treated with medications containing chelating properties or those with high plasma concentrations of H_2_O_2_ and non-transferrin bound iron.

## 1. Introduction

Plant derived polyphenolic compounds exhibit a wide range of antioxidant properties, which have been the subject of considerable research activity in recent years. These antioxidant properties can act as radical scavengers, terminators of free radical chain reactions and chelators of redox active metal ions [1]. Their antioxidant capacity is contingent on their molecular structure and has been shown to be more effective antioxidants in vitro than vitamins E and ascorbic acid on a molar basis [2]. Polyphenols may act directly on reactive oxygen species (ROS) or stimulate endogenous defense systems. They can inhibit the ability of myeloperoxidase to oxidize low-density lipoproteins, a potential anti-atherosclerotic effect [3]. Other potential antioxidant effects include the ability to inhibit cyclooxygenases (prostaglandin-endoperoxide synthase), lipoxygenases and NADPH oxidases [3]. Oxidation of polyphenols produces superoxide radicals (O_2_^−•^), hydrogen peroxide (H_2_O_2_) and an intricate composition of semiquinones and quinones all of which are potentially cytotoxic; however, quinone reductase, catechol-*O*-methyltransferase, and other conjugating enzymes limit the formation of such quinones in vivo [3]. Not surprisingly, there has been some effort to understand the pro-oxidant properties of some polyphenols in the presence of transition metals (Cu, Fe) and or in certain concentrations or a high pH accelerating hydroxyl radical (^•^OH) formation and oxidative strand breakage in DNA in vitro [2].

Green tea catechins, epicatechin and epigallocatechin enhanced Cu^+^-H_2_O_2_-mediated DNA damage, while myricetin, protocatechuic acid and epigallocatechin-3-gallate displayed an antioxidant effect [4]. On the other hand, gallic acid (GA) and epicatechin-3-gallate had both pro-oxidant and antioxidant activity depending on the concentration under the conditions of this in vitro model [4]. Aqueous and ethyl acetate extracts of rooibos leaves (used for redbush tea preparation) as well as dihydrochalcone aspalathin and tannin enhanced deoxyribose (a sugar component of DNA) oxidative degradation by the ^•^OH generating system Fe^3+^-EDTA-H_2_O_2_ [5]. In addition, numerous phenolic acids revealed a dual effect on ^•^OH (Fe^2+^-H_2_O_2_-EDTA system) mediated deoxyribose damage depending on the time of incubation: in the first several seconds, they inhibited and then later revealed an enhancing (pro-oxidant) effect [6]. Conversely, red wine polyphenols: resveratrol, quercetin, and caffeic acid inhibited deoxyribose degradation by two ^•^OH generating systems: Fe^3+^-H_2_O_2_-ascorbate, and Fe^3+^-EDTA-H_2_O_2_-ascorbate [7]. In addition, polyphenol rich extracts of *Moringa oleifera Lam* (*Moringaceae*) inhibited DNA strand breaks induced by a Fe^3+^-H_2_O_2_-ascorbate system [8]. Moreover, the majority of the tested Mauritian endemic plant extracts revealed a protective effect on deoxyribose against the Fenton system supplemented with ascorbic acid [9]. These examples clearly show that polyphenols in general have a protective effect against Fenton systems supplemented with ascorbic acid, while, in Fenton systems without ascorbic acid, the situation is more complex, and these compounds can act as pro-oxidants or antioxidants and can have dual activity depending on their concentration in the reaction mixture [10,11,12]. Therefore, in this study, we investigated the effect of thirteen selected plant phenolics on deoxyribose oxidation induced by the Fenton system composed of Fe^2+^-H_2_O_2_-EDTA. Furthermore, the relationship between the phenolic pro-oxidant/antioxidant effect, their molecular structure and the ability to reduce Fe^3+^ ions into Fe^2+^ (ferric-reducing ability power—FRAP) was analyzed.

## 2. Results

### 2.1. Validity of Experimental Conditions

Incubation of deoxyribose with incomplete (Fe^2+^-EDTA) and complete (Fe^2+^-EDTA-H_2_O_2_) Fenton systems and subsequent boiling with trichloroacetic acid (TCA) and thiobarbituric acid (TBA) solutions resulted in the formation of a chromogen, which gave rise to a sample absorbance at 532 nm (A_532_). As expected, an Fe^2+^-EDTA-H_2_O_2_-induced rise of A_532_ was many-times higher than that caused by an incomplete Fenton system. On the other hand, baseline A_532_ of deoxyribose alone was lower than in the case of deoxyribose with Fe^2+^-EDTA. This may result from the presence of trace amounts of peroxides in water [13]. Phenolics alone and in combination with deoxyribose did not induce formation of additional chromogen. In addition, incubation of all tested polyphenols with H_2_O_2_ did not result in the formation of oxidation products increasing A_532_. All of the afore-mentioned relationships were observed in all series of experiments on the effect of phenolics on the oxidative degradation of deoxyribose; furthermore, they ensured a high validity of obtained results. DMSO as a potent ^•^OH scavenger [14] inhibited deoxyribose oxidation; accordingly, this activity was taken into account in the calculation of the antioxidant or pro-oxidant effect of polyphenols.

### 2.2. Inhibitory Effect of Polyphenols on the Oxidative Degradation of Deoxyribose by the Fenton System

The ability to inhibit the oxidative degradation of deoxyribose by a ^•^OH generating system (Fe^2+^-EDTA-H_2_O_2_) was revealed by five of the 13 tested polyphenols in vitro (Table 1). Four out of the five inhibiting compounds (3,4-dihydroxycinnamic acid (3,4-DCA), 4-hydroxybenzoic acid (4-HBA), 3,4-dihydroxyhydrocinnamic acid ((3,4-DHCA) and catechin) significantly protected deoxyribose from oxidative degradation. This protection ranged from 54.4% ± 28.6% for 3,4-DCA to 38.5% ± 10.4% for catechin; however, no statistically significant differences between the compounds were observed. Furthermore, although the mean inhibition of deoxyribose oxidation by chlorogenic acid (CA) was 7.5%, this did not reach statistical significance (*p* > 0.05).

### 2.3. Enhancing Effect of Polyphenols on the Oxidative Degradation of Deoxyribose by the Fenton System

Seven polyphenols enhanced the oxidative degradation of deoxyribose under the Fe^2+^-EDTA-H_2_O_2_ system (Table 2). The highest pro-oxidative effect resulting in a mean enhancement of deoxyribose oxidation by 95.0% and 60.6% was observed for quercetin and phloridzin, respectively. Ferulic acid (FA) was without significant pro-oxidant activity (Table 2). Ascorbic acid, at a concentration of 10 μmol/L (as a standard of pro-oxidant activity) [15], enhanced the oxidative degradation of deoxyribose by 195.7% ± 19.5% (*n* = 6) under these conditions. Moreover, at the same concentration as the studied polyphenols, two other compounds known as antioxidants: TROLOX**^®^** and *N*-acetylcysteine [16] augmented the Fenton system-induced deoxyribose degradation by 8.1% ± 5.6% and 48.2% ± 31.2% (*p* < 0.05), respectively.

### 2.4. Factors Determining the Pro-Oxidant or Antioxidant Effect of Polyphenols on Deoxyribose Oxidation

**Figure 1:**
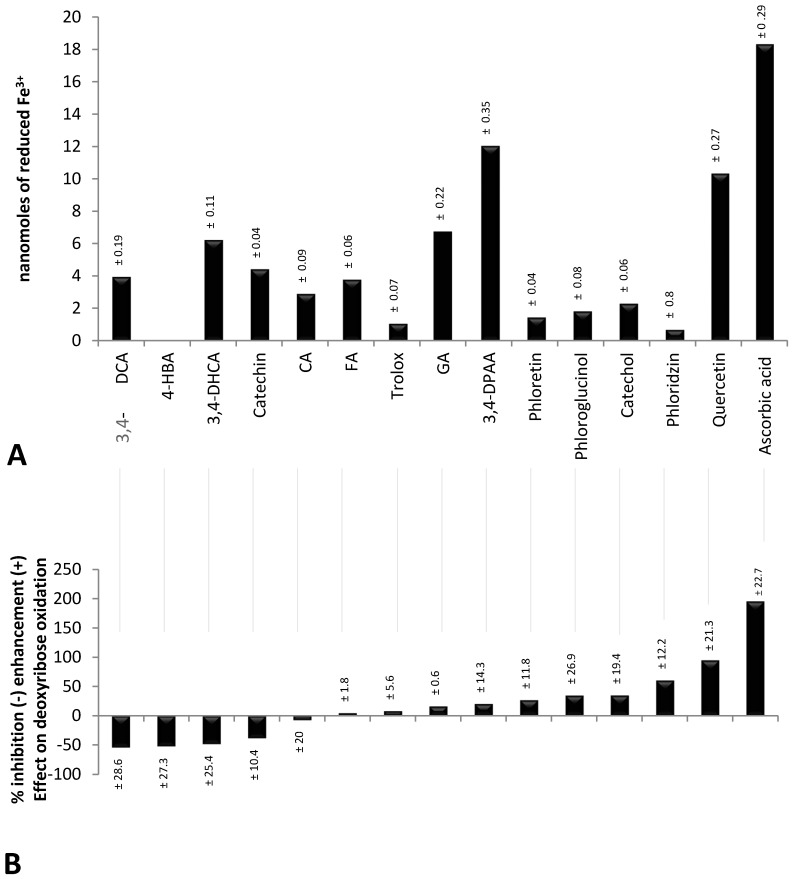
(**A**) nanomoles of reduced Fe^3+^ by polyphenols, TROLOX^®^ and ascorbic acid at a concentration of 5 µmol/L. 4-HBA observed no FRAP at this concentration; (**B**) percentage of inhibition (expressed as a negative value) and enhancement (expressed as a positive value) of deoxyribose oxidation by polyphenols in a concentration of 10 µmol/L. Deoxyribose oxidation was induced by a chemical system Fe^2+^-EDTA-H_2_O_2_; 3,4-DCA—3,4-dihydroxycinnamic acid, 4-HBA—4-hydroxybenzoic acid, 3,4-DHCA—3,4-dihydroxyhydrocinnamic acid, CA—chlorogenic acid, FA—ferulic acid, GA—gallic acid, 3,4-DPAA—3,4-dihydroxyphenylacetic acid, EDTA—ethylenediaminetetraacetic acid.

Figure 1A shows the studied compounds (phenolics, TROLOX^®^ and ascorbic acid) ranked (from left to right) according to their inhibiting (negative value) and enhancing effect (positive value) on the oxidative degradation of deoxyribose by the Fenton system. Below (Figure 1B), their FRAP at a concentration of 5 µmol/L [12] is shown. It clearly delineates that ascorbic acid, which revealed the highest FRAP, had the strongest pro-oxidant effect on deoxyribose oxidation. However, catechin and 3,4-DHCA having distinct FRAP significantly inhibited deoxyribose oxidation. Moreover, quercetin and 3,4-DPAA, the strongest Fe^3+^ reducing agents among the tested phenolics (Figure 1B), enhanced and inhibited deoxyribose oxidation, respectively. A lack of significant correlation between FRAP and the antioxidant/pro-oxidant effect in the group of phenolics (*r* = 0.22, *p* > 0.05) corresponded to the foregoing observations. However, when all compounds were analyzed, a linear correlation was revealed (*r* = 0.65, *p* < 0.001), probably due to the particularly high FRAP and pro-oxidant activity of ascorbic acid.

A multivariate analysis with multiple linear regressions was performed to determine which component of the molecular structure (aliphatic substituent, catechol ring, the number of –OH and –COOH) of polyphenols had a significant effect on the inhibition or enhancement of deoxyribose oxidation (Table 3). In the compounds inhibiting deoxyribose oxidation (Table 1), the correlation between the % inhibition and the number of –OH groups was significant, *r* = 0.58 (*p* = 0.004). The number of –OH groups in the compound molecule accounted for 34.6% of the variance in the inhibitory effect on deoxyribose oxidation (Table 3); however, this has some limitation due to a low number of analyzed compounds. Furthermore, three of the five compounds inhibited deoxyribose oxidation with similar intensity. With the compounds enhancing the oxidation of deoxyribose (Table 2), the number of –OH groups revealed a significant positive correlation *r* = 0.59 (*p* = 0.001) responsible for 32.7% of the variance in pro-oxidant activity (Table 3). This activity also correlated with the occurrence of a catechol structure within the compound molecule (*r* = 0.17, *p* = 0.05) and the presence of an aliphatic substitute at the catechol ring (*r* = −0.22, *p* = 0.04) as estimated with univariate analyses, respectively.

## 3. Discussion

In this study, we evaluated the effect of various polyphenols on the oxidative degradation of deoxyribose through a Fe^2+^-EDTA-H_2_O_2_ system. This system generates ^•^OH via the Fenton reaction (Fe^2+^ + H_2_O_2_ → Fe^3+^ + OH^−^ + ^•^OH) while EDTA chelates Fe ions (Fe^2+^ or Fe^3+^), thereby preventing Fe ions from directly binding to deoxyribose [17,18]. Thus, ^•^OH generated from the reaction of H_2_O_2_ with an Fe^2+^-EDTA complex enter the “free” milieu and can react with deoxyribose and any added scavenger (e.g., polyphenol molecule). The number of Fe^2+^ ions was the limiting factor for ^•^OH production in this Fe^2+^-EDTA-H_2_O_2_ system. Therefore, the addition of any compound that can reduce Fe^3+^ into Fe^2+^ will further stimulate ^•^OH release and subsequent deoxyribose oxidation leading to the rise of sample absorbance at 532 nm. This was noted when ascorbic acid was added to the reaction mixture. Although ascorbic acid itself can scavenge ^•^OH radicals [19], it is a strong Fe^3+^ reducer [12] and thus enhanced deoxyribose degradation about twice as much under the conditions of our experiment. On the other hand, ascorbic acid did not enhance and, in fact, slightly inhibited the aromatic hydroxylation of salicylic acid via the Fe^2+^-EDTA-H_2_O_2_ system [20]. However, in these experiments, the concentration of Fe^2+^ ions and the ratio of Fe^2+^ to H_2_O_2_ were 30 and 10.5 times higher than in our study. Hence, ascorbate-induced regeneration of Fe^2+^ ions (via reduction of Fe^3+^) could have a weak enhancing effect on the generation of ^•^OH radicals. Thus, the scavenging of ^•^OH radicals by ascorbic acid prevailed and resulted in a weak inhibition of salicylic acid hydroxylation.

### 3.1. Plausible Mechanisms of the Anti- or Pro-Oxidant Activity of Polyphenols

While polyphenols can directly react with ^•^OH [2], they also possess distinct FRAP activity [12]. Moreover, iron ions can form complexes with phenolics, which can decrease their reactivity with H_2_O_2_ [2,21]. However, excess EDTA, a strong chelator in the reaction mixture has been shown to prevent the binding of Fe^2+^ and Fe^3+^ to polyphenols [21,22]. Thus, under the conditions of our experiments, the net effect of a tested compound on deoxyribose degradation will be the sum of these two counteracting processes: scavenging of ^•^OH and enhancement of ^•^OH production via regeneration of Fe^2+^ ions. Seven of the thirteen tested polyphenols enhanced deoxyribose oxidation by an Fe^2+^-EDTA-H_2_O_2_ system, while four inhibited this process and two compounds had no significant effect. This differs from previous studies showing significant protection of deoxyribose by polyphenols against ^•^OH-induced degradation [7,8,9]. For instance, quercetin protected deoxyribose degradation from a ^•^OH generating system [7], while, in our experiments; it revealed a significant pro-oxidant effect. In the aforementioned studies, the antioxidant activities of polyphenols or polyphenols containing plant extracts were tested in Fe^3+^-H_2_O_2_-ascorbate or Fe^3+^-EDTA-H_2_O_2_-ascorbate systems [7,8,9]. Since the FRAP of ascorbic acid is stronger than that of phenolics, especially those tested in our study [12], the addition of a polyphenol to a reaction mixture containing Fe^3+^, H_2_O_2_ and ascorbate did not significantly increase the accessibility of Fe^2+^ ions for a reaction with H_2_O_2_ and the subsequent formation of ^•^OH. Thus, the ^•^OH scavenging activity of polyphenols prevailed and was responsible for the protective antioxidant effect under such conditions. In our experiments, ascorbic acid was not used for Fe^2+^ regeneration; however, such capability was evident to some extent by the tested polyphenols. Hence, seven phenolics revealed an enhancing pro-oxidant effect. Accordingly, our results are in line with studies demonstrating the pro-oxidant (enhancing ^•^OH production) activity of polyphenols (e.g., myricetin, quercetin and catechin) in a Fe^3+^-EDTA-H_2_O_2_ system [23,24]. This effect was also observed in the presence of other metal chelators (adenosine diphosphate (ADP), bleomycin) [16,23,25] and disappeared when the ^•^OH generating system was supplemented with ascorbic acid [24]. It is believed that polyphenols as well as other antioxidants (e.g., α-tocopherol, *N*-acetylcysteine) [16] can exert a pro-oxidant effect in systems containing Fe^3+^, H_2_O_2_ and a potent metal chelator that prevent binding of iron to the polyphenol [22]. However, we demonstrated that some phenolics as well as *N*-acetylcysteine and TROLOX^®^ can also act as pro-oxidants in a ^•^OH-generating system composed of Fe^2+^, EDTA and H_2_O_2_. Conversely, four phenolics (3,4-DCA, 4-HBA, 3,4-DHCA and catechin) showed a significant inhibitory effect on the oxidative degradation of deoxyribose through an Fe^2+^-EDTA-H_2_O_2_ system. This is revealing in that, under conditions that unmask and favor pro-oxidant activities, these phenolics behaved as antioxidants most probably due to direct scavenging of ^•^OH.

### 3.2. Determinants of the Polyphenols Effect on Deoxyribose Oxidation by the Fenton System

Although the reduction of Fe^3+^ into Fe^2+^ is supposedly the main mechanism of phenolic pro-oxidant action, under the aforementioned conditions [22], there was no significant correlation between the effect of polyphenols on Fe^2+^-EDTA-H_2_O_2_-induced deoxyribose oxidation and their FRAP. This suggests the occurrence of other mechanisms responsible for the pro-oxidant activity of polyphenols. Polyphenols can undergo autoxidation especially in the presence of iron ions [26] with subsequent generation of H_2_O_2_ and O_2_^−•^. O_2_^−•^ can reduce Fe^3+^ into Fe^2+^ and thus promote ^•^OH generation. The inhibitory effect of superoxide dismutase on polyphenol (e.g., quercetin) induced acceleration of ^•^OH formation by the Fe^3+^-EDTA-H_2_O_2_ system [23] supports this hypothesis. In addition, the reaction between Fe^2+^ and H_2_O_2_ can also produce ferryl ion (FeO^2+^) with an oxidizing potential that degrades deoxyribose to thiobarbituric acid reactive products [27]. On the other hand, the polyphenols’ FRAP was measured at a pH of 3.6 [12], while the effect on deoxyribose oxidation measured at a pH of 7.4. Moreover, the first variable was obtained for the concentration of phenolics at 5 µmol/L while the second was at 10 µmol/L. However, the FRAP of some polyphenols were linear within a wide range (up to 50 µmol/L for quercetin, GA or catechin), and the abovementioned reasons may account for the negative results seen in the correlation analysis. The protective effect of polyphenols correlated with the number of –OH substitutions in the backbone structure. It is possible that ^•^OH can grab a hydrogen atom from one of the hydroxyl groups at the phenolic ring to form water and a less reactive and more stable radical. This corresponds well with a previous report demonstrating an intensification of the ^•^OH scavenging activity of flavonoids with an increased number of –OH substituted in an aromatic B-ring [28]. Furthermore, hydroxyl groups (catechol group, –OH substitutions at position 3, 5, 7 and 4′) are critical for the effective scavenging of peroxynitrite by flavonoids [29], and the inhibition of total ROS generation in kidney homogenates by these compounds intensifies as the number of total –OH groups in their structure increases [30]. Previously, we demonstrated that the presence of a catechol group positively correlated with the ability of phenolics to reduce Fe^3+^ to Fe^2+^ [12]. In this process, both hydroxyl groups of catechol donate two electrons simultaneously losing two hydrogen atoms that results in the formation of a benzoquinone [12]. It is possible that other –OH substitutions could be electron donors for Fe^3+^ reduction [18]. Analysis of the antioxidant activity of eugenol (a phenolic compound and the main component of clove oil) derivatives obtained by the acylation and alkylation of the phenolic hydroxyl group underscores the important role of –OH as a hydrogen atom donor. Four of the 16 derivatives had efficient antioxidant properties; however, such properties were lost with the remaining 12 compounds [31]. Nonetheless, these results propose the presence of other antioxidant mechanisms in addition to those involving –OH substitutions in the phenolic ring [31], which may be responsible for the previously discussed lack of correlation between the effect of phenolics under the Fenton system-induced oxidation of deoxyribose and their FRAP. Since the reduction of Fe^3+^ into Fe^2+^ maintained a high generation of ^•^OH under the Fe^2+^-EDTA-H_2_O_2_ system, this may well explain the positive correlation between the enhanced oxidative degradation of deoxyribose, the presence of a catechol group and the total number of –OH substitutions in phenolics with pro-oxidant activities. Hydroxyl groups can directly scavenge ^•^OH radicals [18,28] and also (especially those present in the catechol structure) reduce Fe^3+^ ions [12]. Both of these activities are recognized as measures of antioxidant potential [32,33]. However, under the Fe^2+^-EDTA-H_2_O_2_ system, the first activity decreased the number of ^•^OH, thus acting as a protectant against the oxidative degradation of deoxyribose, and the second activity enhanced ^•^OH production operating in fact as a pro-oxidant. These activities may explain the seemingly contradictory dual role of these compounds: positive correlation between the total number of –OH substitutions and the pro-oxidant and antioxidant properties of phenolics, respectively. Aliphatic substitutes (e.g., –CH_3_, –CH_2_–CH_3_) in the catechol ring can increase the ability of its –OH groups to be a superior donor of electrons, thus intensifying the potential to reduce Fe^3+^ ions [34]. The negative correlation between the presence of an aliphatic substitute and pro-oxidant phenolic activity is in contrast with the preceding data, thus its elucidation requires further studies.

### 3.3. Applicability to In Vivo Conditions

H_2_O_2_ is detectable in circulating blood and its concentration in human plasma can even reach 80 µmol/L in some pathological conditions [35]. In plasma, the vast majority of iron is bound to transferrin, thus, in healthy subjects, the non-transferrin bound iron that can be involved in ROS generation does not exceed 1 µmol/L. However, this pool of iron can be higher than 15 µmol/L in patients with end stage renal insufficiency or cancer patients undergoing chemotherapy [36]. In healthy subjects, the plasma concentration of phenolics (e.g., 3,4-DCA, 3,4-DHCA, and 3,4-DPAA) ranged between 0.1 to 6 µmol/L [37]. Irrespective of its clinical efficacy, chelation therapy with disodium EDTA has long been used to treat atherosclerotic coronary and peripheral artery disease. It was estimated that roughly 100,000 patients in the United States alone underwent such therapy in 2007 [38]. The plasma EDTA levels in patients that underwent chelation therapy ranged from 0.2 to 1 mmol/L [39]. In all, these studies demonstrate that a reaction between Fe^2+^-EDTA-H_2_O_2_ and selected phenolics can occur in circulating blood in humans and alter ^•^OH generation. Although the concentration of reagents (especially H_2_O_2_) used in our in vitro experiments differ from those found in vivo, a study showing essential acute oxidative injury to circulating lipids, proteins and leukocyte DNA after the addition of ascorbic acid to a standard chelation therapy cocktail containing EDTA in male and female subjects [40] substantiates the applicability of this hypothesis. On the other hand, in healthy subjects, the interaction of ingested polyphenols with iron ions and H_2_O_2_ is more probable. In this case, polyphenols can form complexes with Fe^2+^ and change its reactivity with H_2_O_2_. Thus, the net effect of polyphenols on ^•^OH generation will depend on at least three factors: direct ^•^OH scavenging, reduction of Fe^3+^ ions to Fe^2+^ promoting ^•^OH generation and the formation of polyphenol-iron complexes with an increased or decreased potential to partially reduce H_2_O_2_ to ^•^OH.

## 4. Materials and Methods

### 4.1. Reagents

Ascorbic acid, dimethyl sulfoxide (DMSO), FeSO_4_, TROLOX^®^ (a water-soluble analog of vitamin E), TBA, TCA, disodium EDTA, *N*-acetylcysteine, H_2_O_2_ 30% solution (*w*/*w*) and 2-deoxy-d-ribose were purchased from Sigma-Aldrich Chemical (St. Louis, MO, USA). Sterile phosphate buffered saline (PBS, pH 7.4, without Ca^2+^ and Mg^2+^; osmolarity 300 mOsmol/L) was obtained from Biomed (Lublin, Poland). The following plant polyphenols and their metabolites of the highest purity available acquired from Sigma-Aldrich Chemie GmbH (Steinheim, Germany) or from Fluka, Sigma-Aldrich (Buchs, Steinheim, Germany) were tested: catechin, catechol, CA, 3,4-DCA, 3,4-dihydroxyhydrocinnamic acid (3,4-DHCA), 3,4-dihydroxyphenylacetic acid (3,4-DPAA), FA, GA, 4-hydroxybenzoic acid (4-HBA), phloretin, phloridzin, phloroglucinol and quercetin. Sterile deionized pyrogen-free H_2_O (resistance > 18 MΩ·cm, HPLC H_2_O Purification System, USF Elga, Buckinghamshire, UK) was used throughout the study. Polyphenols were dissolved in DMSO to a final concentration of 100 mmol/L and stored in 20 μL aliquots at −80 °C prior to the assay for no more than 2 weeks. Preparation of the working solution of polyphenol: 10 µL of thawed 100 mmol/L polyphenol stock solution in DMSO was mixed with 990 µL of PBS. Then, 500 µL of this solution was mixed with 500 µL of PBS. The resulting working solution had a polyphenol concentration of 0.5 mmol/L and was used in experiments with deoxyribose oxidation. Control solution of DMSO was prepared in the same way: 10 µL of pure DMSO was mixed with 990 µL of PBS, afterwards 500 µL of this solution was mixed with 500 µL of PBS and subsequently used as a control solvent of polyphenols. Aqueous solution (10 mmol/L) of ascorbic acid, TROLOX^®^ and *N*-acetylcysteine were prepared just before the assay.

### 4.2. Determining the Effect of Plant Phenolics on the Process of Oxidative Degradation of Deoxyribose in the Fenton System

Antioxidant or pro-oxidant properties of polyphenols and their metabolites were examined in the presence of an ^•^OH generating system that subsequently induced oxidative degradation of deoxyribose. ^•^OH was generated at 37 °C in air-exposed incubation PBS buffer (pH = 7.4) by the following system: 10 µmol/L Fe^2+^, 20 µmol/L EDTA and 280 µmol/L H_2_O_2_ (final concentrations in the 500 µL volume of reaction mixture containing 10 mmol/L deoxyribose) [10]. The mechanism for the generation of ROS damaging deoxyribose in the experimental model was based on the Fenton reaction, where intensely reactive ^•^OH are produced through the reaction of H_2_O_2_ with Fe^2+^ ions [11]. Phenolics and the other compounds were tested at a final concentration of 10 µmol/L. The experimental and control systems were prepared simultaneously by adding to a 460 µL deoxyribose solution in PBS with the 10 µL volumes of working solutions of polyphenol, FeSO_4_, EDTA, and H_2_O_2_. Some negative control samples (blank) contained H_2_O instead of H_2_O_2_. Some additional controls received a control solution of DMSO instead of a working polyphenol solution (Table 4). Samples were incubated for 10 min at 37 °C. This incubation time was chosen based on our preliminary experiments, which resulted in a mere oxidation of approximately 30% of deoxyribose molecules in the assayed sample [10]. Therefore, under these conditions, it was possible to study the inhibitory (antioxidant) or enhancing (pro-oxidant) effect of polyphenols on deoxyribose oxidation. After incubation, all samples were mixed with 0.5 mL 60 g/L TCA and 0.25 mL TBA solution (1 g TBA in 100 mL of 0.05 N NaOH), boiled for 20 min, and then their absorbance at 532 nm (A532) was measured against a blank containing deoxyribose alone [10]. The polyphenol antioxidant or pro-oxidant effect was expressed as % inhibition or % enhancement of oxidative degradation of deoxyribose by a complete Fenton system (Fe^2+^-EDTA-H_2_O_2_). Results were obtained from six series of separate experiments with each tested compound and were corrected for the antioxidant activity of DMSO present in the working polyphenol solutions.

### 4.3. Statistical Analysis

Results (% inhibition or % enhancement of deoxyribose oxidation) were expressed as mean (standard deviation) and median. The effect of polyphenols on the oxidative degradation of deoxyribose (comparison between deoxyribose in the Fenton system and deoxyribose with polyphenol in the Fenton system) was analyzed with Mann–Whitney U test. To determine the statistical difference within the group of antioxidant (inhibiting deoxyribose oxidation) and pro-oxidant (enhancing deoxyribose oxidation) phenolics, a Kurskal–Wallis non-parametric one-way ANOVA was performed with an appropriate Bonferroni correction post hoc test.

In order to determine the molecular structures contributing to the effect of polyphenols on deoxyribose oxidation, a multivariate analysis was carried out with a multiple linear regression, using the backward stepwise technique. The percent of inhibition or enhancement of deoxyribose oxidation in the presence of polyphenols at a concentration of 10 µmol/L were the dependent variables, and the independent variables included the number of –OH and carboxyl (–COOH) substitutions in the backbone structure, the existence of a catechol structure within the compound molecule, and the presence of an aliphatic substitute at a catechol ring. The relationship between the effects of polyphenols on deoxyribose oxidation, and their FRAP was assessed with Pearson’s correlation. FRAP values of polyphenols were taken from our previously published study [12]. In all cases, a *p*-value of <0.05 was considered significant. Prior to commencing the study, ethical clearance and study protocol was accepted by the Ethics Committee of the Medical University of Lodz.

## 5. Conclusions

Seven of the 13 tested phenolics enhanced ^•^OH generation while the remaining six inhibited or had no significant effect on ^•^OH activity under the Fe^2+^-EDTA-H_2_O_2_ system, which favors the unmasking of pro-oxidant effects in the investigated compounds. Since this system can occur in the blood of patients that have undergone chelation therapy, the possibility of some ingested dietary polyphenols enhancing ^•^OH production and inducing oxidative damage to various circulating biomolecules may be envisaged.

Fruits and vegetables contain a variety of polyphenols including those enhancing and inhibiting ^•^OH production under the Fe^2+^-EDTA-H_2_O_2_ system. It is possible that, in vivo, their pro- and antioxidant action counteract; therefore, the risk of an induction of oxidative stress via a high intake of dietary polyphenols seem negligible. On the other hand, selective supplementation of the diet with phenolics exhibiting pro-oxidant activity under the Fe^2+^-EDTA-H_2_O_2_ system in vitro may increase the possibility of systemic oxidative stress in patients treated with EDTA or other medications with chelating properties (e.g., bleomycin) or those with high plasma concentrations of H_2_O_2_ and non-transferrin bound iron. However, confirmation of this hypothesis requires further clinical studies.

## Figures and Tables

**Table 1:** Inhibitory (antioxidant) effect of polyphenols on the oxidative degradation of deoxyribose by the system (Fe^2+^-EDTA-H_2_O_2_).

Polyphenol ^†^	Chemical Structure	Inhibition of Deoxyribose Oxidation (%)
3,4-Dihydroxycinnamic acid	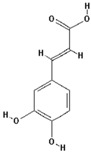	54.4 ± 28.6 (62.8) *
–OH: 3		
Catechol: 1		
–COOH: 1		
Aliphatic Substitute at the Catechol Ring: 1		
4-Hydroxybenzoic acid	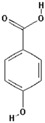	52.4 ± 27.3 (56.3) *
–OH: 2		
Catechol: 0		
–COOH: 1		
Aliphatic Substitute at the Catechol Ring: 0		
3,4-Dihydroxyhydrocinnamic acid	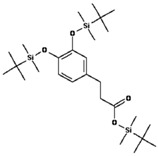	48.5 ± 25.4 (52.5) *
–OH: 3		
Catechol: 1		
–COOH: 1		
Aliphatic Substitute at the Catechol Ring: 1		
Catechin	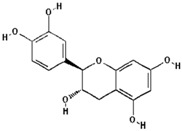	38.5 ± 10.4 (44.4) *
–OH: 5		
Catechol: 1		
–COOH: 0		
Aliphatic Substitute at the Catechol Ring: 0		
Chlorogenic acid	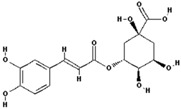	7.5 ± 20 (0.01)
–OH: 6		
Catechol: 1		
–COOH: 1		
Aliphatic Substitute at the Catechol Ring: 1		

^†^ selected molecular structures and their number present in the studied phenolics are listed below the name of the compound. In addition, 10 mmol/L of deoxyribose in phosphate buffer (pH = 7.4) was incubated under the Fenton system (10 µmol/L Fe^2+^—20 µmol/L ethylenediaminetetraacetic acid (EDTA)—280 µmol/L H_2_O_2_) with and without the addition of 10 µmol/L of polyphenol for 10 min at 37 °C. Afterwards, samples were mixed with 0.5 mL 60 g/L of trichloroacetic acid (TCA) and 0.25 mL of thiobarbituric acid (TBA) solution (1 g TBA in 100 mL of 0.05 N NaOH), boiled for 20 min for chromogen development and subsequent measurement of absorbance at 532 nm. Results obtained from six series of experiments were expressed as mean ± standard deviation (median). * significant inhibition vs. deoxyribose alone incubated with (Fe^2+^-EDTA-H_2_O_2_).

**Table 2:** Enhancing (pro-oxidant) effect of polyphenols on the oxidative degradation of deoxyribose by the Fenton system (Fe^2+^- ethylenediaminetetraacetic acid (EDTA)-H_2_O_2_).

Polyphenol ^†^	Chemical Structure	Enhancement of Deoxyribose Oxidation (%)
Ferulic acid	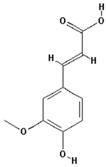	4.6 ± 1.8 (4.3)
–OH: 2		
Catechol: 0		
–COOH: 1		
Aliphatic Substitute at the Catechol Ring: 0		
Gallic acid	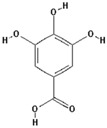	16.1 ± 0.6 (15.2) *
–OH: 4		
Catechol: 2		
–COOH: 1		
Aliphatic Substitute at the Catechol Ring: 0		
3,4-Dihydroxyphenylacetic acid	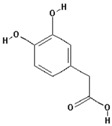	20.3 ± 14.3 (13.7) *
–OH: 3		
Catechol: 1		
–COOH: 1		
Aliphatic Substitute at the Catechol Ring: 1		
Phloretin	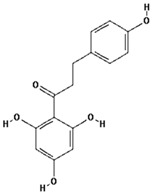	26.9 ± 11.8 (33.3) *
–OH: 4		
Catechol: 0		
–COOH: 0		
Aliphatic Substitute at the Catechol Ring: 0		
Phloroglucinol	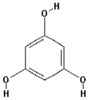	34.8 ± 26.9 (30.8) *
–OH: 3		
Catechol: 0		
–COOH: 0		
Aliphatic Substitute at the Catechol Ring: 0		
Catechol	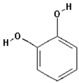	34.9 ± 19.4 (38.1) *
–OH: 2		
Catechol: 1		
–COOH: 0		
Aliphatic Substitute at Catechol Ring: 0		
Phloridzin	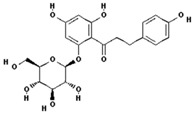	60.6 ± 12.2 (55.7) *
–OH: 7		
Catechol: 0		
–COOH: 0		
Aliphatic Substitute at the Catechol Ring: 0		
Quercetin	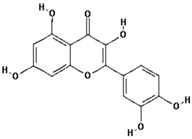	95.0 ± 21.3 (97.5) *
–OH: 5		
Catechol: 1		
–COOH: 0		
Aliphatic Substitute at the Catechol Ring: 0		

^†^ selected molecular structures and their number present in the studied phenolics are listed below the name of the compound. In addition, 10 mmol/L deoxyribose in phosphate buffer (pH = 7.4) was incubated with the Fenton system (10 µmol/L Fe^2+^—20 µmol/L EDTA—280 µmol/L H_2_O_2_) with and without 10 µmol/L of polyphenol for 10 min at 37 °C. Other details are the same as for Table 1. Results obtained from six series of experiments were expressed as mean ± standard deviation (median). * Significant enhancement vs. deoxyribose alone incubated with (Fe^2+^-EDTA-H_2_O_2_).

**Table 3:** Factors influencing the anti-oxidant and pro-oxidant properties of studied plant phenolics—a summary of multivariate regression.

Dependent Variable	Independent Variables	Entry into Model	Multiple *r*	Squared Multiple *r*	*p*	Zero Order *r*
Inhibition of deoxyribose oxidation (*n* = 5)	Catechol ring	Out				0.177
	Aliphatic substitute	In				
	–OH substitutions	In	0.588	0.346	0.004	0.570
	–COOH substitute	In				−0.053
Enhancement of deoxyribose oxidation (*n* = 8)	Aliphatic substitute	Out				−0.22
	–OH substitutions	In	0.572	0.327	0.001	0.590
	–COOH substitute	In				
	Catechol ring	In				−0.166

The independent variables were the number of –OH and –COOH substitutions in the backbone structure, the occurrence of a catechol structure within the compound molecule, and the occurrence of an aliphatic substitute at a catechol ring.

**Table 4:** Design of experiments on the effect of polyphenols on the oxidative degradation of deoxyribose in the Fenton system (Fe^2+^-EDTA-H_2_O_2_).

No.	Sample	Volumes of Working Solutions of Reagents and Tested Polyphenols (µL)						
		A	B	C	D	E	F	G
		Deoxyribose	Polyphenol	DMSO	FeSO_4_	EDTA	H_2_O	H_2_O_2_
1	Blank	460	-	-	-	-	40	-
2	Positive	460	-	-	10	10	10	10
3	Polyphenol effect	460	10	-	10	10	-	10
4	DMSO control *	460	-	10	10	10	-	10
	**Additional Controls**							
5	Incomplete system **	460	-	-	10	10	20	-
6	Deoxyribose with polyphenol ^†^	460	10	-	-	-	30	-
7	Polyphenol alone ^††^	-	10	-	-	-	490	-
8	Polyphenol with H_2_O_2_^†††^	-	10	-	-	-	480	10

Working solutions were mixed in alphabetical order. A—10.9 mmol/L deoxyribose in sterile phosphate buffered saline (PBS) (pH = 7.4); B—0.5 mmol/L polyphenol in PBS with addition of 70 mmol/L DMSO; C—70 mmol/L DMSO in PBS; D—0.5 mmol/L aqueous solution of FeSO_4_; E—1 mmol/L aqueous solution of EDTA; G—14 mmol/L H_2_O_2_; * control of polyphenol solvent; ** incomplete Fenton system without H_2_O_2_; ^†^ and ^††^ control for possible formation of a colored complex from polyphenol and deoxyribose or polyphenol alone. ^†††^ control for possible formation of a polyphenol oxidation product that may increase sample absorbance at 532 nm.

## Appendix A—Chemical Compounds Studied in This Article

3,4-Dihydroxycinnamic acid (PubChem CID: 689043); 4-Hydroxybenzoic acid (PubChem CID: 135); 3,4-Dihydroxyhydrocinnamic acid (PubChem CID: 91697097);

Catechin (PubChem CID: 9064); Chlorogenic acid (PubChem CID: 1794427); Ferulic acid (PubChem CID: 445858); Gallic acid (PubChem CID: 370); 3,4-Dihydroxyphenylacetic acid (PubChem CID: 547); Phloretin (PubChem CID: 4788); Phloroglucinol (PubChem CID: 359); Catechol (PubChem CID: 289); Phloridzin (PubChem CID: 6072); Quercetin (PubChem CID: 5280343).
